# Epidemic investigation of benign prostatic obstruction with coexisting overactive bladder in Shanghai Pudong New Area and its impact on the health-related quality of life

**DOI:** 10.1186/s12894-019-0513-1

**Published:** 2019-09-03

**Authors:** Qing-Tong Yi, Min Gong, Chu-Hong Chen, Wei Hu, Ru-Jian Zhu

**Affiliations:** Department of Urology, Shanghai Pudong Hospital, Fudan University Pudong Medical Center, Shanghai, China

**Keywords:** Benign prostatic obstruction, Epidemic investigation, Health-related quality of life, Overactive bladder

## Abstract

**Background:**

We aimed to investigate the prevalence, relative risk factors, and the impact on the health-related quality of life (HRQoL) of benign prostatic obstruction (BPO) with coexisting overactive bladder (OAB) in men aged over 50 and living in Shanghai Pudong New Area.

**Methods:**

Using a multi-stage sampling and descriptive epidemiological method, 1632 men were selected from among the general population. Participants completed an evaluation of lower urinary tracts symptoms (LUTS), including international prostate symptom score (IPSS) and quality of life (QoL) questionnaires. Erectile function was assessed using the International Index of Erectile Function-5 (IIEF-5) questionnaire. In addition, the Overactive Bladder Symptom Score (OABSS) and King’s health questionnaire (KHQ) were used to assess the impact of BPO with coexisting OAB on the HRQoL. Maximum flow rate (Q_max_), postvoid residual urine volume (PVR) and prostate-specific antigen (PSA) were also recorded.

**Results:**

A total of 1476 men with complete data were analyzed. The overall prevalence of BPO with coexisting OAB was 39.6%. Age and prostate volume were associated risk factors for BPO with coexisting OAB. In addition, BPO with coexisting OAB negatively impacted the HRQoL, with increased IPSS, QoL, OABSS, and KHQ scores and decreased IIEF-5 scores compared to that in patients with BPO without OAB.

**Conclusions:**

Q_max_, PVR and serum PSA did not predict whether the patients had a combined BPO + OAB or not. The prostate volume and age were associated risk factors for BPO with coexisting OAB. BPO is a progressive disease and may be one of the risk factors for OAB.

## Background

Benign prostatic obstruction (BPO) is one of the most common urinary disorders leading to lower urinary tracts symptoms (LUTS) in middle-aged and elderly men. LUTS can be divided into storage, voiding and post-micturition symptoms. Overactive bladder (OAB) features urinary urgency, usually with frequency and nocturia, and with or without urge urinary incontinence [1, 2]. Currently, the relationship between BPO and OAB remains unclear, and large epidemic investigations of BPO with coexisting OAB are lacking worldwide. Therefore, we performed a sample survey of men aged ≥50 years in Shanghai Pudong New Area, to investigate the prevalence of BPO with coexisting OAB. In addition, we investigated the associated risk factors and the impact of BPO with coexisting OAB on the health-related quality of life (HRQoL).

BPO commonly coexists with OAB and consequently decreases the QoL in men. The following factors should be considered in the study of patients with lower urinary tract function: observations of patients (symptoms), quantification of symptoms, physician’s observations (anatomy, function and compliance), QoL measures, and socioeconomic evaluations [3].

Patient-reported outcomes (PROs) refer to any report from the patient regarding their health and treatment status. It includes reports of patient symptoms, physical, psychological and social functional status, healthy behavior, different tendencies expressed by patients for different treatments, a desire to participate (or not participate) in a treatment, patient satisfaction with treatment, doctor-patient communication and cooperative treatment [4]. PROs can accurately reflect the subjective feelings of patients with OAB, and can be used to assess the impact of OAB on the QoL of patients and treatment response [5]. BPO and OAB questionnaires, including international prostate symptom score (IPSS), QoL, Overactive Bladder Symptom Score (OABSS) and OAB scale are commonly used in clinical practice. The IPSS and OABSS mainly reflect changes in the symptoms of LUTS, while the QoL reflects the degree of suffering. The OABSS scoring table includes four OAB-related symptom issues (urinary urgency, frequency, nocturia and urge urinary incontinence) and the total score is sum these individual item scores [6]. Furthermore, the OABSS score is highly correlated with the IPSS score.

The HRQoL is an assessment of how an individual’s well-being may be affected over time by a disease, disability or disorder. The current concept of the HRQoL acknowledges that individuals place their actual situation in relation to their personal expectations. King’s health questionnaire (KHQ), a kind of PROs, has high validity and reliability and is now widely accepted as a useful instrument for evaluating the HRQoL of OAB patients [7]. KHQ consists of two subscales: HRQoL and lower urinary symptoms severity. HRQoL consists of nine domains: general health, incontinence impact and seven limitations including role, physical, social, personal, emotion, sleep/energy, and severity measures. Lower urinary symptoms severity scale contains ten questions and the higher the score, the more severe the symptoms [8]. In the present study, the WHO-QOL cross-cultural quality of life questionnaire translation method was used to translate the original KHQ into Chinese [9, 10].

Participants were asked to complete questionnaires which mentioned above regarding personal information, urination, sexual function, and the QoL.

## Methods

### Subjects and sampling methods

This survey was a cross-sectional study conducted from October 2012 to September 2016. Using standard formula, we calculated the sample size based on a two-sided t-test with a significance level of 5%. According to the total prevalence rate of benign prostatic hyperplasia (BPH) in males over 50 years old in Pudong New Area (62.9%) [11], we calculated the sample size to be 1434. Permanent residents aged 50 years or more from 10 subdistricts/towns in Pudong New Area who met BPO diagnostic criteria were included. A multi-stage stratified random sampling was used to select the geographic regions; three-stage sampling including district-subdistrict, town-committee or village was used. Ten subdistricts or towns in Pudong New Area were randomly selected; four neighborhood or village committees were then randomly selected from each selected subdistrict or town. Patients diagnosed with BPO were chosen according to the residents’ health records at the community health centers. The neighborhood/village committee, as a unit, informed the selected patients of the study. Selected patients with voluntary participation were finally enrolled.

The inclusion criteria were as follows meanwhile: the presence of voiding or/and post-micturition symptoms, prostate volume ≥ 25 mL on transrectal ultrasonography, maximum urinary flow rate (Qmax) ≤15 mL/s. A series of tests were conducted in the community health centers, including a medical history inquiry, physical examination, urinalysis, prostate-specific antigen (PSA) assessment, imaging of the urinary system, and uroflowmetry, to exclude other disorders that could cause LUTS (e.g., neurogenic bladder dysfunction, detrusor overactivity, detrusor sphincter dyssynergia, foreign body in the bladder or urethra, bladder neoplasms, prostatic neoplasms, vesical calculus, bladder neck contracture, small capacity bladder, urethral stricture, chronic pelvic pain syndrome, urinary tract infection, etc.). Exclusion criteria included patients of BPO with no symptoms, a post-void residual urine volume of more than 50 mL, severe mental disorder (except anxiety or depression), previous prostate or bladder surgery, and the use of medications affecting urination.

### Inquiry content and data collection

In addition to the clinical examination, participants completed a field questionnaire survey, which collected data regarding personal information, urination, sexual function, and the QoL. LUTS severity was evaluated using IPSS and QoL questionnaires. IIEF-5 was used to assess penile erectile function. For patients with BPO and coexisting OAB, the OABSS and KHQ were used to assess the impact of OAB on the QoL. Questionnaires were checked to ensure completeness and were supplemented with the examination results.

### Diagnostic criteria of BPO with coexisting OAB

Patients with BPO who had OAB symptoms were defined as BPO with coexisting OAB. The OAB diagnostic criteria were as follows: total OABSS ≥3 and urgent micturition ≥2, with duration of more than 3 months. Based on the IPSS and OABSS, the patients were divided into a mild (IPSS score 0~7 or OABSS score 3~5), moderate (IPSS score 8~19 or OABSS score 6~11), and severe groups (IPSS score 20~35 or OABSS score 12~15) [6, 12].

### Quality control

All investigators were professionally trained. The questionnaires were reviewed twice and 1% of patients were selected for a revisit. The database was built using excel software with double entry and was subjected to verification and logic error checking. When any problems were detected, the original records were checked and the corresponding contents were revised as appropriate.

### Statistics

Statistical analyses were performed using SPSS 18.0 software (SPSS Inc. Chicago). Categorical variables are described as numbers and percentages. Group comparisons were performed using the chi-squared test for classification data and the t-test and analysis of variance for continuous data. For quantitative variables with non-normal distribution, group comparisons were performed using Mann–Whitney U test. Binomial logistic regression analyses were performed to determine the risk factors of BPO with coexisting OAB. *P* value < 0.05 was considered statistically significant.

Ethical approval was given by the medical ethics committee of Shanghai Pudong Hospital and informed consent was obtained from the participants in all cases.

## Results

### General characteristics of the study population and prevalence of BPO with coexisting OAB

A total of 1632 participants were recruited from 10 subdistricts or towns in Shanghai Pudong New Area during October 2012 to September 2016. A complete dataset was achieved in 1476 participants; 156 participants were dropped due to incomplete information. A total of 697 participants lived in cities, while 779 lived in the suburbs. General characteristics of the study population were shown in Tables 1 and 2. The prostate volume, the IPSS and QOL scores were significantly higher, and the IIEF-5 score was significantly lower, in patients with BPO and coexisting OAB compared to those in patients without coexisting OAB(*P* < 0.05) (Table 1).

**Table 1:** General characteristics of the study population

Variable	BPO without OAB (*n* = 892)	BPO with coexisting OAB (*n* = 584)	*P* value
Prostate volume (mL)	55.6 ± 20.7	62.7 ± 26.5	0.005
Serum PSA (ng/mL)	3.6 ± 0.8	3.7 ± 0.7	0.624
Testosterone (nmol/L)	14.2 ± 4.6	13.9 ± 4.3	0.196
Qmax (mL/s)	10.2 ± 3.9	10.1 ± 3.8	0.162
PVR (mL)	19.8 ± 17.3	20.5 ± 18.7	0.820
BMI (kg/m2)	24.3 ± 3.7	24.6 ± 4.1	0.461
IPSS (score)	19.2 ± 6.8	23.5 ± 7.3	0.000
QoL (score)	3.9 ± 0.9	4.6 ± 1.2	0.000
IIEF-5 (score)	9.9 ± 2.3	9.0 ± 1.7	0.036

*BPO* Benign prostatic obstruction, *OAB* Overactive bladder syndrome, *PSA* Prostate-specific antigen, *Qmax* Maximum flow rate, *PVR* Postvoid residual urine volume, *IPSS* International prostate symptom score, *QoL* Quality of life, *IIEF-5* International index of erectile function-5, *OABSS* Overactive bladder symptom score

**Table 2:** Prevalence of BPO with coexisting OAB

Group	Sample size (n)	BPO with coexisting OAB (n)	Prevalence (%)	OR	*P* Value
Age (years)					
50~	288	81	28.1	1	
60~	363	124	34.2	1.33	0.142
70~	545	243	44.6	2.10	0.002
80~	247	119	48.2	2.37	0.002
90~	33	17	51.5	2.72	0.014
BMI (kg/m^2^)					
< 24	381	167	43.8	1	
24~	337	154	45.7	1.07	0.808
≥ 27	317	152	47.9	1.18	0.552
Diabetes					
No	1255	488	38.9	1	
Yes	221	96	43.4	1.21	0.209
Emotional State					
Normal	1361	521	38.3	1	
Anxiety/ depression	115	63	54.8	1.95	0.036
Total	1476	584			

*BMI* Body mass index, *BPO* Benign prostatic obstruction, *OAB* Overactive bladder, *OR* Odds ratio

The overall prevalence of BPO with coexisting OAB was 39.6% (584/1476) in men over 50 years old in Shanghai Pudong New Area. The prevalence of BPO with coexisting OAB increased with age, with a significantly higher prevalence in the 70–79, 80–89, and ≥ 90 years age groups compared to that in the 50–59 years age group (*P* < 0.05). In addition, a higher prevalence of BPO with coexisting OAB was observed in participants with anxiety-depression compared to that in normal participants (*P* < 0.05). Moreover, an increasing trend in the prevalence of BPO with coexisting OAB was observed in participants with higher BMI or diabetes, though both of which have no significant different (Table 2).

### Comparison among different age groups in BPO with coexisting OAB

IPSS, QoL, OABSS, and KQH scores significantly increased while the IIEF-5 score significantly decreased with increasing age (*P* < 0.05). However, there were no significant differences among different age groups in aspects of prostate volume, serum PSA, testosterone, Qmax, PVR and BMI (*P*>0.05). The QoL items most affected included the general health status, severity of urinary problems, and sleep/energy. In all, the quality of life of patients with BPO with coexisting OAB decreased with increasing age (Table 3).

**Table 3:** Comparisons among different age groups in BPO with coexisting OAB ($\overline{\mathrm{x}}$ ± s)

Age Group	50~years (*n* = 81)	60~years (*n* = 124)	70~years (*n* = 243)	80~year (*n* = 119)	90~years (*n* = 17)
Prostate volume (mL)	57.7 ± 24.1	56.9 ± 16.3	62.8 ± 26.5	69.6 ± 34.1	77.1 ± 20.4
*P* Value		0.214	0.235	0.358	0.347
Serum PSA (ng/mL)	3.2 ± 0.7	3.4 ± 0.6	3.7 ± 0.9	3.9 ± 1.2	4.2 ± 1.1
*P* Value		0.541	0.512	0.336	0.357
Testosterone (nmol/L)	17.2 ± 6.4	16.0 ± 5.7	12.7 ± 5.2	9.2 ± 3.9	7.1 ± 3.2
*P* Value		0.544	0.112	0.054	0.05
Qmax (mL/s)	11.5 ± 4.2	10.1 ± 3.4	10.1 ± 3.8	9.5 ± 4.1	8.8 ± 3.4
*P* Value		0.337	0.463	0.425	0.372
PVR (mL)	10.0 ± 4.2	12.6 ± 5.3	18.4 ± 6.7	26.8 ± 8.2	29.3 ± 8.8
*P* Value		0.130	0.688	0.393	0.246
BMI (kg/m2)	24.3 ± 3.6	25.2 ± 4.0	24.7 ± 4.2	23.9 ± 3.9	25.5 ± 2.3
*P* Value		0.051	0.221	0.768	0.095
IPSS	9.1 ± 3.3	14.3 ± 2.7	18.6 ± 3.9	24.7 ± 5.4	27.8 ± 6.6
*P* Value		0.031	0.028	0.000	0.001
QoL	4.2 ± 0.7	4.4 ± 0.7	4.7 ± 0.9	5.0 ± 1.2	5.4 ± 1.5
*P* Value		0.042	0.040	0.038	0.001
IIEF-5	10.5 ± 1.4	9.2 ± 0.9	7.7 ± 0.7	6.1 ± 0.8	5.6 ± 1.0
*P* Value		0.040	0.003	0.000	0.000
OABSS	7.2 ± 3.1	8.4 ± 3.6	9.1 ± 3.4	10.7 ± 4.0	11.6 ± 3.7
*P* Value		0.046	0.009	0.000	0.000
KHQ	23.4 ± 6.5	23.2 ± 7.3	28.6 ± 9.1	30.0 ± 9.5	28.7 ± 10.6
*P* Value		0.988	0.042	0.028	0.005
General health status	25.6 ± 8.9	25.3 ± 8.4	28.4 ± 9.7	28.6 ± 7.7	27.0 ± 10.6
*P* Value		0.596	0.048	0.000	0.000
Severity of urinary problems	29.4 ± 9.1	29.4 ± 10.2	29.5 ± 9.1	31.3 ± 9.7	26.5 ± 8.9
*P* Value		0.735	0.500	0.548	0.681
Role limitations	18.6 ± 8.8	18.3 ± 6.2	18.2 ± 5.9	19.8 ± 7.7	19.1 ± 7.5
*P* Value		0.996	0.451	0.007	0.633
Physical limitations	3.6 ± 2.0	3.2 ± 2.1	2.9 ± 1.5	3.2 ± 2.6	3.3 ± 1.8
*P* Value		0.793	0.659	0.594	0.771
Social limitations	16.9 ± 7.1	18.3 ± 7.9	19.7 ± 9.5	18.8 ± 9.1	18.9 ± 7.7
*P* Value		0.009	0.048	0.006	0.011
Personal relationships	2.2 ± 0.9	2.3 ± 0.9	2.2 ± 0.9	2.3 ± 1.0	2.2 ± 1.0
*P* Value		0.218	0.500	0.17	0.500
Emotions	8.3 ± 5.1	7.5 ± 3.7	6.9 ± 3.7	9.3 ± 5.3	7.0 ± 2.9
*P* Value		0.633	0.742	0.564	0.530
Sleep/Energy	29.8 ± 11.5	29.0 ± 9.3	37.5 ± 11.3	34.0 ± 9.8	33.5 ± 7.5
*P* Value		0.707	0.000	0.000	0.001
Coping urination problems	14.8 ± 4.8	15.5 ± 6.7	18.4 ± 8.0	15.9 ± 5.1	18.4 ± 6.1
*P* Value		0.467	0.001	0.058	0.000

*BPO* Benign prostatic obstruction, *OAB* Overactive bladder syndrome, *PSA* Prostate-specific antigen, *Q*_*max*_ Maximum flow rate, *PVR* Postvoid residual urine volume, *IPSS* International prostate symptom score, *QoL* Quality of life, *IIEF-5* International index of erectile function-5, *OABSS* Overactive bladder symptom score, *KHQ* King’s health questionnaire. *P* Value vs 50~ years

### Impact of LUTS severity on HRQoL in patients with BPO and coexisting OAB

With increased LUTS severity, IPSS, QoL, OABSS, and KHQ scores increased, while IIEF-5 scores decreased (*P* < 0.05). Thus, the QoL in patients with BPO and coexisting OAB declined with LUTS severity. The QoL items most affected included the general health perception, severity of the micturition problem, and sleep/energy status (Table 4).

**Table 4:** The impact of LUTS severity on the HRQoL in patients with BPO and coexisting OAB ($\overline{x}$ ± s)

Impact on HRQoL (score)	Mild LUTS (*n* = 227)	Moderate LUTS (*n* = 254)	Severe LUTS (*n* = 103)
IPSS	5.4 ± 2.2	14.7 ± 3.5	26.3 ± 6.7
*P* Value		0.000	0.000
QoL	3.3 ± 0.5	4.6 ± 0.7	5.4 ± 1.1
*P* Value		0.000	0.000
IIEF-5	10.5 ± 1.4	7.7 ± .07	5.6 ± 1.0
*P* Value		0.017	0.009
OABSS	3.6 ± 1.3	9.5 ± 3.2	12.8 ± 3.9
*P* Value		0.036	0.012
KHQ	21.7 ± 6.3	26.8 ± 8.5	30.9 ± 11.4
*P* Value		0.001	0.000
General health status	20.4 ± 7.2	25.9 ± 9.5	28.3 ± 10.9
*P* Value		0.002	0.021
Severity of urinary problems	22.5 ± 11.6	27.6 ± 8.5	30.5 ± 9.2
*P* Value		0.001	0.001
Role limitations	13.8 ± 7.9	16.2 ± 8.3	19.9 ± 7.8
*P* Value		0.000	0.000
Physical limitations	2.6 ± 1.9	2.9 ± 1.5	3.3 ± 1.8
*P* Value		0.654	0.728
Social limitations	15.2 ± 7.1	17.8 ± 9.2	19.2 ± 7.9
*P* Value		0.000	0.000
Personal relationships	1.7 ± 0.7	2.3 ± 0.9	2.1 ± 0.9
*P* Value		0.059	0.633
Emotions	7.3 ± 4.6	7.7 ± 3.9	8.9 ± 3.2
*P* Value		0.742	0.684
Sleep/Energy	20.8 ± 7.5	28.3 ± 8.7	33.9 ± 11.6
*P* Value		0.008	0.001
Coping urination problems	12.7 ± 4.4	16.4 ± 8.0	18.8 ± 6.7
*P* Value		0.024	0.018

*BPO* Benign prostatic obstruction, *OAB* Overactive bladder syndrome, *IPSS* International prostate symptom score, *QoL* Quality of life, *IIEF-5* International index of erectile function-5, *OABSS* Overactive bladder symptom score, *KHQ* King’s health questionnaire. *P* Value vs mild LUTS

### Risk factors of BPO with coexisting OAB

Univariate logistic regression analyses revealed that the prevalence of BPO with coexisting OAB increased with the age, prostate volume, PVR and serum PSA (*P* < 0.05). The prevalence of BPO with coexisting OAB was higher in participants with diabetes mellitus. The prevalence of BPO with coexisting OAB was 1.09 times higher in obese participants (47.9%; BMI ≥27 kg/m^2^) compared to that in normal participants (43.8%; BMI < 24 kg/m^2^) (Table 5).

**Table 5:** Univariate regression analysis of risk factors for BPO with coexisting OAB

Variable	B	SE	P	OR	EXP(B)95%CI
Age	0.002	0.006	0.000	1.032	[1.016,1.4321]
Prostate volume	0.215	0.053	0.000	1.013	[1.008,1.375]
Q_max_	0.028	0.129	0.670	0.990	[0.755,1.252]
PVR	0.005	0.005	0.000	1.010	[1.006,1.016]
Serum PSA	0.017	0.104	0.000	1.040	[1.007,1.426]
Testosterone	0.500	0.447	0.650	0.973	[0.253,1.458]
Diabetes	0.025	0.138	0.200	1.213	[0.794,1.357]
BMI Level	0.098	0.109	0.280	1.084	[0.891,1.373]

*Q*_*max*_ Maximum urine flow rate, *PVR* Postvoid residual urine volume, *PSA* Prostate specific antigen, *BMI* Body mass index, *BPO* Benign prostatic obstruction, *OAB* Overactive bladder syndrome, *OR* Odds ratio

A multivariate logistic regression analysis revealed that BPO with existing OAB was associated with the age and prostate volume (*P* < 0.05). With increasing age or prostate volume, LUTS, IPSS, QoL, and OABSS scores increased, while IIEF-5 scores decreased (Table 6).

**Table 6:** Multivariate logistic regression analysis of risk factors for BPO with coexisting OAB

Variable	B	SE	P	OR	EXP(B)95%CI
Age	0.047	0.010	0.000	1.049	[1.012,1.524]
Prostate volume	0.010	0.004	0.011	1.010	[1.003,1.013]
Q_max_	0.019	0.024	0.433	1.019	[0.779,1.318]
BMI	0.023	0.026	0.372	1.023	[0.746,1.425]
Anxiety	0.205	0.370	0.579	1.227	[0.980,1.336]
Diabetes	0.275	0.970	0.354	1.316	[0.825,1.344]

*Q*_*max*_ Maximum urine flow rate, *BMI* Body mass index, *BPO* Benign prostatic obstruction, *OAB* Overactive bladder syndrome. Variable levels: Diabetes: Yes = 1, No = 0; BMI: BMI < 24 kg/m^2^ = 1, 24 ≤ BMI < 27 kg/m^2^ = 2, ≥27 kg/m^2^ = 3. *SE* Standard error, *OR* Odds ratio

## Discussion

LUTS have traditionally been related to bladder outlet obstruction (BOO), which is often caused by benign prostatic enlargement (BPE) resulting from the histologic condition of BPH. BPO and OAB are common clinical and public health concerns, causing male urinary dysfunction and affecting the QoL, whether occurring alone or in combination [2, 13, 14]. OAB occurs in 11.8% of adults over 18 years old, and LUTS morbidity and severity increase along with age [15]. The symptoms of OAB are the most bothersome symptoms in patients with LUTS induced by BPH and the effect of LUTS during the urine storage period is greater than that during the urination period [16]. In addition, BPH-related LUTS are one of the main reasons for a decline in patient QoL, and are associated with increased prostate volume which leads to BOO [13, 14]. It has been reported that 30 to 60% of cases with BOO caused by BPO will appear as OAB symptoms, and 30 to 40% of cases of BOO combined with OAB still show up as OAB symptoms after the release of BOO [17]. Moreover, OAB morbidity is positively correlated with the degree of BOO [18]. Thus, BPH, OAB, and LUTS are closely related to each other, and all are positively correlated with age.

At present, large-sample epidemiological studies of the prevalence of BPO with coexisting OAB and related factors are lacking. In our study, an epidemiological survey of the BPO population aged ≥50 years in Pudong New Area was conducted using a multistage, stratified, random sampling method to better understand the prevalence of BPO with coexisting OAB, the related risk factors and its impact on the QoL. The results demonstrated that the overall prevalence of BPO with coexisting OAB was 39.6% (584/1476), close to previous reports [18]. Interestingly, we found a higher prevalence of BPO with coexisting OAB in participants with anxiety-depression. This suggested that we should pay attention to the mental health of patients and necessary psychological interventions could be performed to treat patients with anxiety and depression to improve overall efficacy. Furthermore, those who had diabetes or obesity had an increasing trend in the prevalence of BPO with coexisting OAB, indicating that reducing weight and controlling blood sugar may help prevent and reduce the occurrence of BPO with coexisting OAB.

Previously reported risk factors for the clinical progression of BPH include age, serum PSA, prostate volume, Q_max_, PVR, IPSS, chronic inflammation of the prostate, metabolic syndrome, intravesical prostatic protrusion, prostate transition zone volume, and transition zone index [19–25]. The etiology of OAB and its correlation with BPO remains unclear. There are many factors affecting the occurrence and development of BPH, OAB and LUTS. The present results showed that IPSS, QoL scores, prostate volume, PVR and LUTS increased with increasing age in patients with BPO, while the Q_max_ and IIEF-5 score decreased. In addition, BMI and IPSS and QoL scores were higher while IIEF-5 scores were lower, in patients with BPO and coexisting OAB compared to that in patients with BPO only. In contrast, there was no significant difference in prostate volume, Q_max_, PVR, serum PSA, and testosterone levels between the two groups. These findings indicated that Q_max_, PVR and serum PSA did not predict whether the patients had a combined BPO + OAB or not. In addition, the prostate volume and age were associated risk factors for BPO with coexisting OAB. Thus, we believe that BPO is a progressive disease and may be one of the risk factors for OAB.

The present results showed that the QoL items affected by BPO with coexisting OAB most were those regarding the general health status, the severity of the urinary problems, and sleep/energy. With an increase in LUTS severity, the IPSS, QoL, OABSS and KHQ scores increased, while the Q_max_ and IIEF-5 scores decreased. Age and prostate volume are not only risk factors for BPO with coexisting OAB, but are also for sexual dysfunction. Moreover, LUTS affects sexual function, including sexual desire, erectile function, ejaculation status and sexual satisfaction, with a positive correlation between the severity of LUTS and sexual dysfunction [26]. Thus, BPO, OAB, and LUTS are independent risk factors for sexual dysfunction. Among LUTS, those with the most impact on the sexual function of patients with BPO, were nocturnal polyuria, dysuria and frequent micturition.

The treatment goal of BPO with coexisting OAB is to improve the clinical symptoms and QoL. All men with LUTS should be formally assessed prior to any allocation of treatment in order to establish symptom severity classification. Treatment can be tailored according to the severity of disease and individualized for cost-effective management [27].

There were several limitations in the study. There is evidence to suggest that prostate volume less than 25 ml can still have significant obstruction and intravesical prostatic protrusion is a better and more reliable predictor of BPO [28, 29]. However, we did not record this data. The samples included cannot cover all patients (sampling bias) and a systematic error due to distortions or incompleteness in memory may exist (memory bias).

## Conclusions

In summary, Q_max_, PVR and serum PSA did not predict whether the patients had a combined BPO + OAB or not. The prostate volume and age were associated risk factors for BPO with coexisting OAB. BPO is a progressive disease and may be one of the risk factors for OAB. All men with LUTS should be formally assessed and treatment can be tailored according to the symptom severity classification.

## Data Availability

The datasets used and/or analyzed during the current study are available from the corresponding author on reasonable request.

## Abbreviations

BOO: Bladder outlet obstruction
BPE: Benign prostatic enlargement
BPH: Benign prostatic hyperplasia
BPO: Benign prostatic obstruction
HRQoL: Health-related quality of life
IIEF-5: International Index of Erectile Function-5
IPSS: International prostate symptom score
KHQ: King’s health questionnaire
LUTS: Lower urinary tracts symptoms
OAB: Overactive bladder
OABSS: Overactive Bladder Symptom Score
PROs: Patient-reported outcomes
PSA: Prostate-specific antigen
PVR: Postvoid residual urine volume
Q_max_: Maximum flow rate
